# Incorporation of MRI-AIF information for improved kinetic modelling of dynamic PET data

**DOI:** 10.1186/2197-7364-1-S1-A43

**Published:** 2014-07-29

**Authors:** Hasan Sari, Kjell Erlandsson, Kris Thielemans, David Atkinson, Simon Arridge, Sebastien Ourselin, Brian Hutton

**Affiliations:** Institute of Nuclear Medicine, University College London, London, UK; Center for Medical Imaging, University College London, London, UK; Center for Medical Image Computing, University College London, London, UK

Pharmacokinetic analysis of dynamic PET data requires estimation of the arterial input function (AIF). An alternative method to collecting blood samples during scanning is the simultaneous estimation approach in which AIF and kinetic parameters are estimated at once [1]. We have investigated how information from a simultaneous MRI-AIF can be incorporated into this model and evaluated its effect on estimated kinetic parameters by comparing absolute bias and coefficient of variation (CV) values on influx constants (Ki). As the delivery of the tracer or contrast agent is dominated by vascular flow dynamics, PET-AIF and MRI-AIF boluses will have similar peak shapes if the same injection speed is used [2, 3]. Hence it can be assumed that the values of λ_1_ of eq. 1 [1] can be fixed to reduce the number of parameters in fitting. Simulated data was generated using two tissue compartment model with rate constants from a clinical brain study [5]. Noise was added using eq. 2 where α set to 0.5,2 and 4. AIF scaling was done using one blood sample obtained at the end of the study, or by fixing A_3_ and λ_3_ to their true values assuming they can be determined using multiple blood samples.12

**Figure 1:**
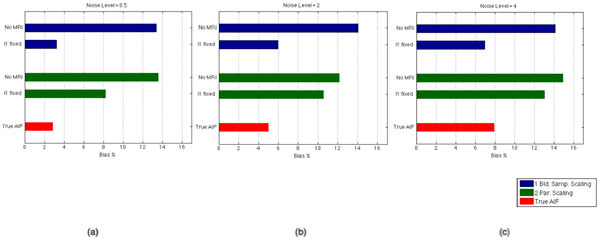
Percentage absolute bias on Ki estimates, averaged over three ROIs, when noise level was set to (a) 0.5, (b) 2, (c) 4. Blue bars show the bias with the scaling method with one blood sample at the end of the study, green bars show the bias with the scaling method where the tail is fit to the multiple blood samples towards the end of study and red bars show the bias which would be present when all six parameters of the AIF was set to their true values.

**Figure 2:**
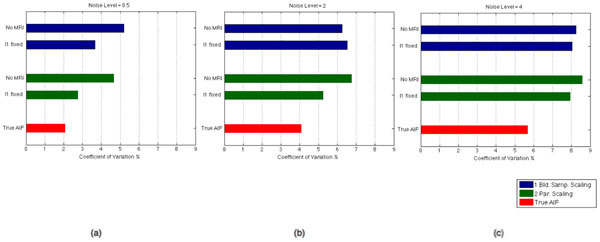
Coefficient of variation values over 50 Ki estimates, averaged over three ROIs, when noise level was set to (a) 0.5, (b) 2, (c) 4. Blue bars show the CV with the scaling method with one blood sample at the end of the study, green bars show the CV with the scaling method where the tail is fit to the multiple blood samples towards the end of study and red bars show the CV which would be present when all six parameters of the AIF was set to their true values.

As shown in Figure 1 and 2, when λ_1_ is fixed, larger improvements (from 13.84% to 5.38%, averaged on all noise levels) were seen on the bias of estimates with one blood sample compared to the method with multiple samples (from 13.54% to 10.60%). For CV, improvement was slightly higher on scaling with multiple samples when λ_1_ was fixed.

We have shown that information from simultaneous MRI-AIF can reduce the bias on K_i_ estimates and multiple blood samples might not be necessary, as the scaling method with one blood sample performed better when a parameter reflecting the early part of the MRI-AIF is included.
